# Domestication Enhances the Plastic Response of Root Economic Traits to Soil Variation in the Perennial Crop *Cnidoscolus aconitifolius*

**DOI:** 10.3390/plants15182850

**Published:** 2026-09-18

**Authors:** Jazmin A. López-Herrejón, Rocío Vega-Frutis, Miguel A. Munguía-Rosas

**Affiliations:** 1 Laboratorio de Ecología Terrestre, Departamento de Ecología Humana, Cinvestav, Km 6 Antigua Carretera a Progreso, Mérida 97310, Mexico; jazmin.lopez@cinvestav.mx; 2 Programa Académico de Biología, Unidad Académica de Agricultura, Universidad Autónoma de Nayarit, Km. 9 Carretera Tepic-Compostela, Xalisco 63780, Mexico; rocio.vega@uan.edu.mx

**Keywords:** domestication, phenotypic plasticity, roots, root average diameter, root economic spectrum, root economics space, root nitrogen content

## Abstract

Domestication-driven shifts toward resource acquisition are well-documented above ground, but how it impacts belowground strategies remains controversial. Furthermore, the role of environmental variation and phenotypic plasticity in shaping root economic space traits has been largely neglected. Here, the effects of domestication, soil origin, and their interaction on four root economic traits (average root diameter, specific root length, root tissue density, and root nitrogen content) were experimentally assessed using wild and domesticated chaya (*Cnidoscolus aconitifolius*) grown in forest, home garden, and intensive orchard soils. Domestication did not trigger a unidirectional shift to more acquisitive roots, but significantly enhanced phenotypic plasticity across soils. Significant interactions for average root diameter and nitrogen content showed that domesticated plants exhibited variable, decoupled responses in agroecosystem soils, while domesticated and wild plants remained very similar in forest soils. Wild plants remained phenotypically canalized. Multivariate analysis revealed that expected trade-offs among traits and orthogonality between conservation and collaboration gradients were only partially met. In conclusion, belowground strategies in this perennial crop are reconfigured by domestication through increased phenotypic plasticity, allowing independent modulation of key root traits, particularly under nutrient-rich environments.

## 1. Introduction

A central tenet in functional ecology is that variation in ecological strategies for acquiring vs. retaining resources is driven by a resource acquisition–conservation trade-off operating coordinately across plant organs [1,2,3]. Although this theoretical framework has provided valuable, testable predictions, empirical evidence for a whole-plant economics spectrum remains mixed; while some studies show coordination between above- and below-ground organs [1,4], others indicate decoupled strategies [5,6]. Recent research suggests that this decoupling occurs in part because, unlike leaves, roots do not follow a simple, one-dimensional conservation gradient [7]. Instead, root resource economics operates within a bi-dimensional space (Root Economics Space, RES) driven by two orthogonal gradients: (i) a conservation gradient, anchored at opposite ends by plants with roots of high tissue density (RTD, a proxy for resource conservation) vs. high nitrogen content (RN, a proxy for nutrient acquisition); and (ii) a collaboration gradient, bounded by plants with high average root diameter (AD; positively correlated with dependence on arbuscular mycorrhizal fungi [AMF]) vs. high specific root length (SRL; positively correlated with autonomous resource exploitation and the number of meristematic root cells) [7,8].

Plant resource-use strategies are also strongly influenced by the environment [9,10]. Predictable and coordinated shifts in the resource economics of above- and below-ground organs are expected across environmental gradients; specifically, a conservative strategy is predicted in plants growing in resource-poor habitats, whereas an acquisitive strategy is expected in those inhabiting resource-rich environments [9,10]. When environmentally induced variation in traits associated with a resource-use strategy occurs within the same species, it is mainly driven by phenotypic plasticity [11,12]. Since roots are often more plastic than aerial organs [12,13], such differences may not only explain cases of decoupled above- and belowground resource-use strategies, but also environmentally induced shifts that plants may have in their position within the RES [14,15].

Human activities have also induced shifts in resource-use strategies of plants throughout domestication [16]. These shifts stem from the interplay between selective processes (i.e., artificial selection) and environmentally induced phenotypic variation [17,18]. Since domesticated plants frequently inhabit nutrient-rich environments, they often exhibit more acquisitive strategies than their wild relatives in aerial organs [16,19]. Therefore, this shift is at least partially explained by phenotypic plasticity [16,20]. However, this pattern is far less clear in belowground organs [21,22,23]. Experiments comparing domesticated plant species (predominantly seed-propagated annual crops) with their wild relatives under controlled conditions have found poor support—showing either no effect or idiosyncratic responses to domestication—for a shift toward more acquisitive roots in domesticated plants [21,22,23,24,25]. Although economic root traits are known to be plastic and responsive to local soil conditions [26,27] and domestication may alter both plastic responses [28] and phenotypic integration [29], the relative influence of domestication per se vs. phenotypic plasticity on RES traits has rarely been addressed [30,31]. If domesticated plants also have higher phenotypic plasticity and reduced phenotypic integration of roots, it may confer greater versatility in how root economic traits respond to soil variation.

The goal of this study was to evaluate the effect of domestication, soil origin, and their interaction on the four core traits of the bi-dimensional RES (AD, SRL, RN, RTD). The study species was chaya (*Cnidoscolus aconitifolius*), a clonally propagated, perennial leafy vegetable. Since its origin, the domesticated form of chaya has traditionally been cultivated by the Maya in home gardens under organic fertilization [32,33], while it has recently been introduced into intensively managed orchards utilizing synthetic fertilizers; hence, the most nutrient-rich soil is expected in the latter habitat [33]. Because wild plants naturally occur in the forest, soil from this habitat was used as a reference baseline. Therefore, chaya serves as an ideal model system to evaluate how domestication per se impacts core RES traits and how this effect interacts with soil origin. Given that domesticated chaya exhibits more acquisitive leaves than its wild relatives [33], under the whole-plant economics framework [1,2,3], one may expect the domesticated form to display inherently more acquisitive roots than its wild progenitor (i.e., high RN and low RTD). However, if phenotypic plasticity has also been altered by domestication—rendering domesticated plants more responsive to nutrient availability than their wild ancestors [12,17]—a strong interaction between domestication and soil origin is expected. Consequently, considering that mycorrhizal associations are typically more profitable under resource limitation [34], a shift toward an acquisitive (high RN; low RTD) and “do-it-yourself” (high SRL; low AD) strategy were predicted for domesticated plants when grown in nutrient-rich soils. Conversely, wild plants were expected to exhibit a conservative (high RTD; low RN) and collaborative (high AD; low SRL) strategy that remains more canalized across soil origins.

## 2. Results

### 2.1. Root Trait Variation

**Figure 1:**
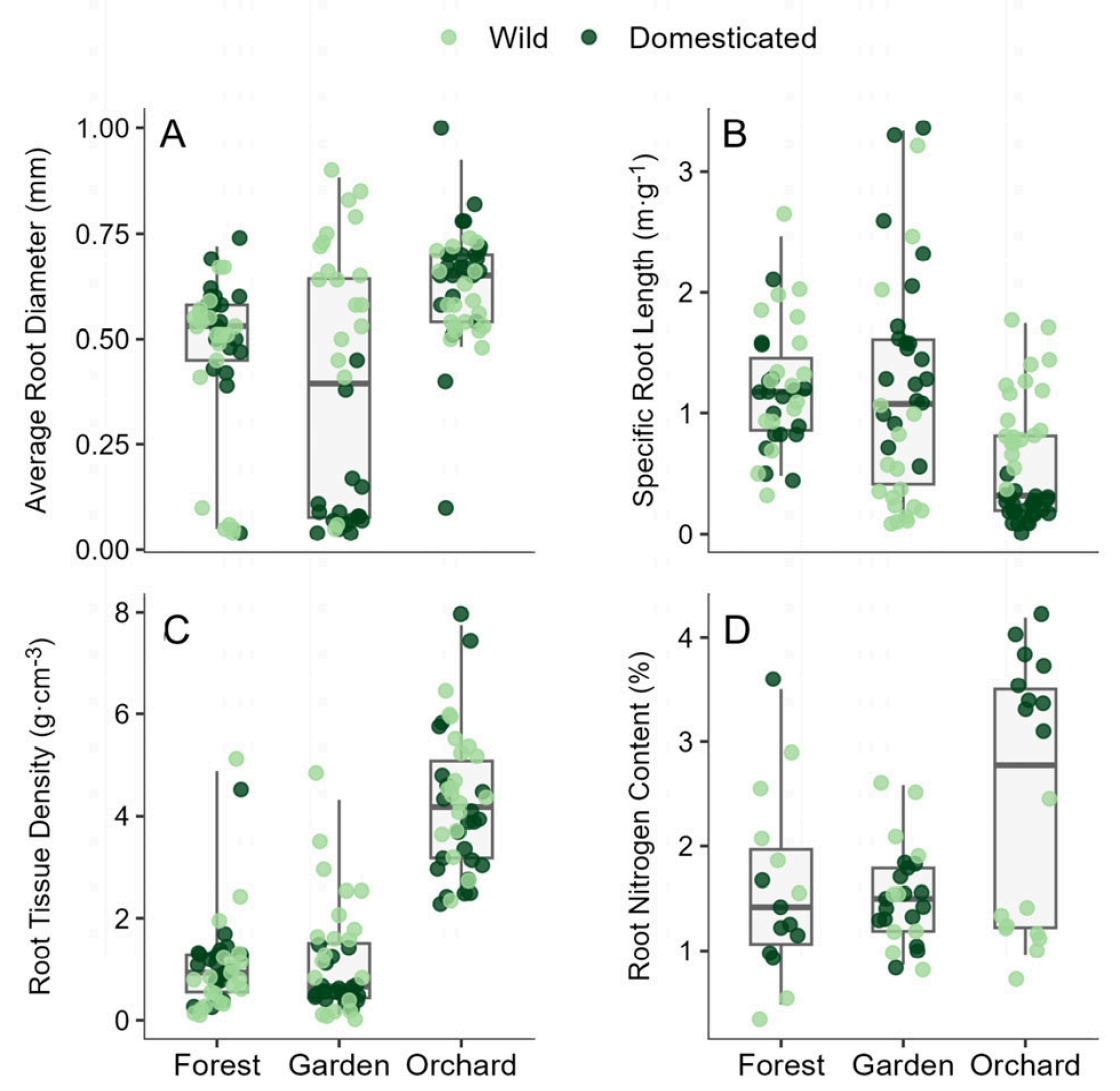
Boxplots of four functional root traits associated with the root economics space: (**A**) Average root diameter, (**B**) specific root length, (**C**) root tissue density and (**D**) root nitrogen content of chaya plants (*Cnidoscolus aconitifolius*) grown in soils of three different origins: forest patches (Forest), home gardens (Garden) and intensive orchards (Orchard). Dots indicate individual wild (light green) and domesticated (dark green) plants. In all cases, the boxes indicate the interquartile range, the internal line represents the median, and whiskers extend to the minimum and maximum values excluding outliers (interquartile range × 1.5). N = 138 plants. Mass units (g) in plot C refer to grams of dry mass (see Materials and Methods for details).

In general, with the exception of SRL, all measured root traits exhibited wider variation in domesticated plants (CV range: 63.38–387.41%) than in their wild counterparts (CV range: 43.05–256.30%) (Figure 1; Table 1). The magnitude of this difference was contingent upon the specific trait. Specifically, RN, AD, and RTD were 1.47, 1.72 and 3.03 times more variable in domesticated plants than in their wild counterparts (Table 1). Conversely, the variation in SRL was 2.06 times greater in wild than in domesticated plants (Table 1). Root trait variation depended on the specific combination of plant variety and soil origin (Figure 1; Table 1). For instance, wild plants exhibited their highest variation for AD, SRL, and RTD in home garden soils, whereas domesticated plants expressed their maximum variation for these same traits under contrasting soil conditions—specifically in home garden soil for AD, in forest soil for SRL and in orchard soil for RTD (Table 1). Furthermore, while domestication restricted the variation in SRL across all soil origins, it drastically amplified the variation in RTD (up to 11-fold) specifically when grown in orchard soil (Table 1). In contrast, RN showed an opposite pattern: domestication severely restricted its variation in managed environments (home garden and orchard soils) but amplified it nearly two-fold in forest soil (Table 1).

### 2.2. Effects of Domestication and Soil Origin on Root Traits

**Figure 2:**
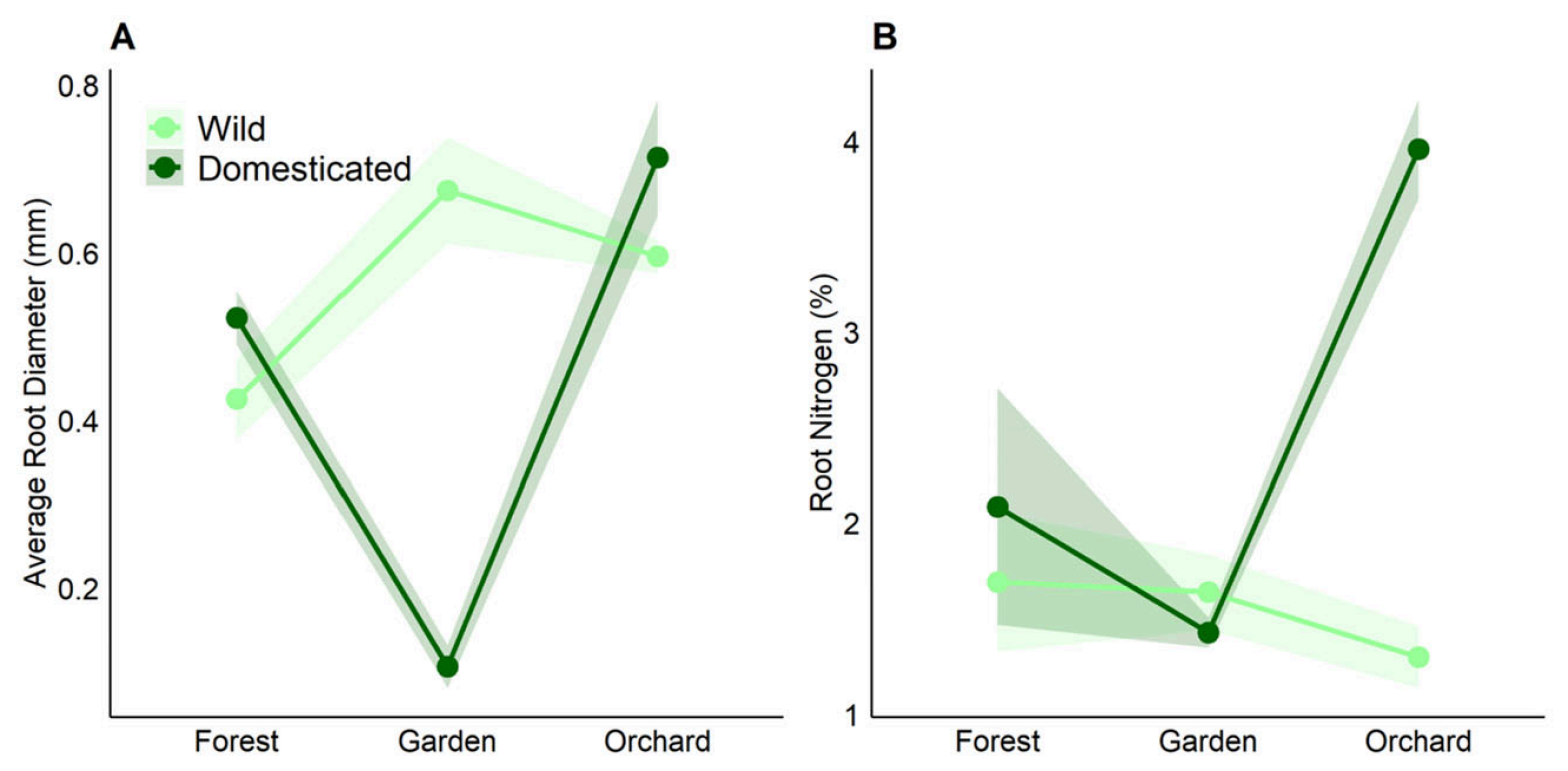
Interaction plots showing the effects of domestication (wild vs. domesticated plants) × soil origin (forest, home garden [Garden], intensive orchards [Orchard]) on average root diameter (**A**) and root nitrogen content (**B**) in chaya (*Cnidoscolus aconitifolius*). In all cases, circles represent mean values, and shaded areas indicate ± 1 SE of the mean. N = 138 plants.

Among the four root traits considered in this study, significant differences between domesticated and wild plants were detected only for AD (Table 2). Specifically, wild plants had an AD 1.19 times larger than domesticated plants (Table 3). Significant differences among soil origins were detected for AD and SRL (Table 2). Specifically, AD and SRL exhibited the highest values in orchard soils, whereas values for these two traits were similar between forest and home garden soils (Table 3). Finally, a significant domestication x soil origin interaction was found for AD and RN, and this term explained the largest proportion of deviance in the models (≈80% of AD deviance and ≈90% of RN deviance; Table 2). For AD, wild and domesticated plants showed similar values in forest and orchard soils, but there was a dramatic difference when grown in home garden soil. On the other hand, RN was similar between wild and domesticated plants in forest and home garden soils, but radically different in orchard soil (Table 3; Figure 2).

The plasticity index (PI, calculated as: Maximum mean-minimum mean/maximum mean.) was consistently larger in domesticated than in wild plants; however, the size of the difference (ΔPI = 0.41–0.48) was far higher for AD and RN (Table 3). Finally, the contribution of the random factor (genetic family) to the explained deviance was only minor (<2.1%) for all traits, except SRL, where it explained 15.14% (Table 2).

### 2.3. Multivariate Ordination

**Figure 3:**
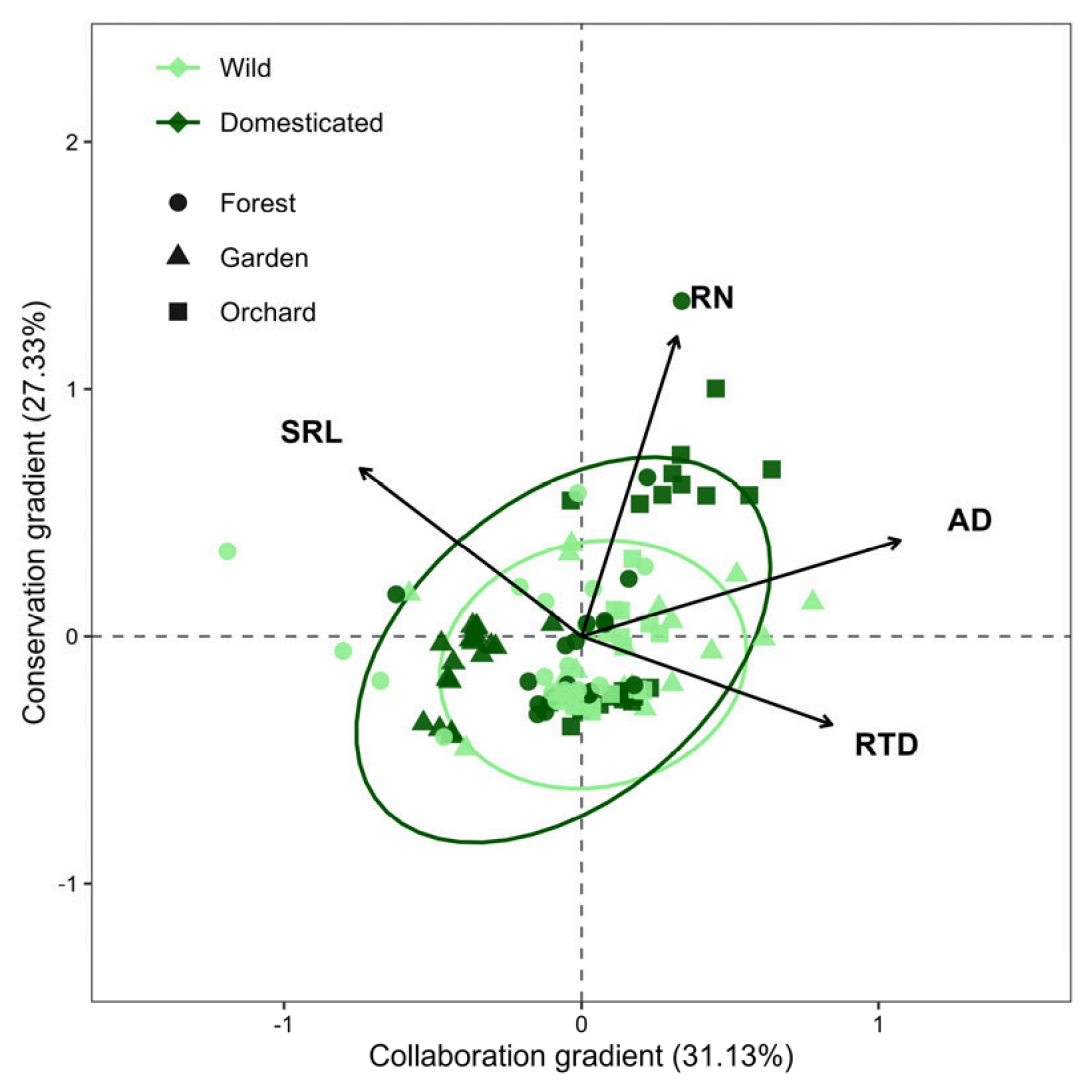
Ordination plots showing PC1 and PC2 obtained through a principal component analysis for four root economics traits: average root diameter (AD), specific root length (SRL), root tissue density (RTD) and root nitrogen content (RN). PC1 tentatively represents a collaboration gradient, and PC2 a conservation gradient. Different colors indicate two varieties of chaya (*Cnidoscolus aconitifolius*: wild vs. domesticated plants) and different symbols indicate three different soil origins: forest, home gardens (Garden) and intensive orchards (Orchard). 99% confidence ellipses are also shown.

According to the principal component analysis, the first (PC1) and the second (PC2) principal components explained 31.13% and 27.33% of the variance, respectively (cumulative explained variance = 58.46%). In absolute values, AD, RTD, and SRL exhibited the largest loadings on PC1; the first two had positive loadings (AD = 0.61; RTD = 0.53), whereas the last had a negative loading (SRL = −0.48). On the other hand, RN (0.81) and SRL (0.46) had the largest positive loadings on PC2, while RTD was the only trait with a negative loading on this axis (−0.24). The PCA ordination plot showed a putative collaboration gradient along PC1, with AD in the positive range and SRL in the negative range, and a putative conservation gradient along PC2, with RN in the positive range and RTD in the negative range (Figure 3). The interior angle between the loadings of the traits associated with PC1 (AD and SRL) was 114.57°, whereas the angle between traits associated with PC2 (RTD and RN) was 106.12° (Figure 3). The interior angles between the remaining loadings were 54.91°for AD and RN, 59.62° for RN and SRL, 159.90° for SRL and RTD, and 45.41° for RTD and AD (Figure 3).

In the multivariate space, an extensive overlap was observed between plant varieties; however, the space occupied by domesticated plants was larger than that of wild plants (Figure 3). Furthermore, the multivariate distribution of wild and domesticated plants followed different distribution patterns across soil origins. In forest soil, both varieties overlapped extensively near the center of the ordination space. In contrast, wild and domesticated plants exhibited separate distributions in home garden soil; that is, wild plants were mainly distributed in the positive end of PC1, whereas domesticated plants were distributed toward the negative end of the same axis. Finally, orchard soil positioned both varieties toward the upper-right quadrant; however, this trend was far more pronounced in domesticated than in wild plants, with the former occupying the extreme positive ends where values of RN are high (Figure 3).

## 3. Discussion

According to the results, although domestication as a single factor did not exert a significant effect on most root economic traits, clear variation patterns were observed when wild and domesticated plants were grown in soils of contrasting origin. Interestingly, domestication increased the plastic response of root economic traits to soil variation in chaya. Specifically, larger coefficients of variation and plasticity indices were observed in domesticated plants for almost all evaluated traits, whereas these traits were largely canalized in wild plants. Furthermore, as predicted, a significant domestication × soil origin interaction was exhibited by key root economic traits. Specifically, marked differences were shown by AD and RN under the more favorable soil conditions of agroecosystems, whereas similar values were exhibited by both plant varieties in forest soil, where less advantageous conditions were anticipated due to the lack of fertilization. On the other hand, the existence of a bi-dimensional RES was only partially supported by the ordination of the values for the four root economic traits. While, as expected, opposite signs were displayed by traits associated with the collaboration (AD and SRL) and conservation gradients (RN and RTD), their orthogonality was not supported by the analysis of the internal angles of loading vectors which revealed unexpected covariation patterns among traits. Consistent with the idea that phenotypic plasticity has been increased by domestication, a broader area in the multivariate space was occupied by domesticated plants. Despite this wide overlap, a partial separation between wild and domesticated plants was observed in the ordination, but only in the regions occupied by agroecosystem soils. All these results suggest that domestication, via enhanced phenotypic plasticity—together with the previously reported domestication-driven reduction in phenotypic integration [29]—allows root economic traits to be modified independently under favorable soil conditions to maximize resource acquisition.

A significant main effect of domestication was detected only for AD, with domesticated chaya plants displaying lower values. A similar effect of domestication on AD was observed in hexaploid wheat (*Triticum aestivum*) [35], which contrasts with a study in maize (*Zea* spp.) where domestication increased values of the same trait [36]. The lack of a domestication effect on SRL, RTD, and RN aligns with previous multi-species studies [21,23]. However, it contrasts with a study on weeping meadow grass (*Microlaena stipoides*), in which domestication significantly decreased SRL and increased RTD [25]. These contrasting findings confirm the previously identified idiosyncratic effect of domestication on root economic traits [21,22,23].

While univariate analyses revealed significant effects of domestication and soil origin on AD, these effects were not additive. Specifically, in home garden soil, domesticated plants exhibited a dramatic reduction in AD, while no difference between wild and domesticated plants was seen in the other two soil origins. Since AD is often positively associated with AMF colonization that helps the plant to obtain limiting nutrients (mainly phosphorus) [34,37] and home garden soil possessed higher bioavailable phosphorus content in the study area [33], the observed reduction in AD of domesticated plants in this agroecosystem is likely a plastic response to a phosphorus-rich environment. Thinner roots are not only cheaper to construct but are also more efficient at resource exploitation and more sensitive to environmental conditions [14,38]. Similarly, a non-additive effect of domestication and soil origin was found for RN. Specifically, a greater RN was observed in domesticated relative to wild plants in the soil of the most intensively managed agroecosystem, while no difference was found between plant varieties in the other origins. Again, this is likely due to plastic response to intensive soil management that includes the use of synthetic nitrogen fertilizers such as anhydrous ammonia and urea ammonium nitrate [33], which can be absorbed by the plant and translocated to different plant organs and in this way, increase metabolic investment and finally resource acquisition [39]. The existence of these non-additive effects of domestication and soil origin together with greater plasticity indices and CVs in domesticated than in wild plants strongly suggests that, as expected, domestication has increased phenotypic plasticity in key root economic traits. Although in the literature, the effect of domestication on phenotypic plasticity is highly contingent upon the crop species, the specific trait being measured, and the environmental variable considered, current research remains highly biased toward above-ground organs [28,40]. Consequently, these findings contribute to expanding the small number of available studies that have identified that domestication increases phenotypic plasticity in root economic traits [31,41]. Further studies are clearly warranted to confirm whether this positive effect of domestication on the phenotypic plasticity of root economic traits is generalizable. In particular, more studies on perennial crops are needed, as they have received far less attention.

An important idea behind the RES is the existence of ecological trade-offs between root traits associated with opposite strategies within a given gradient [7,8]. Since root economic traits belong to a single plant phenotype, trade-offs are often assessed through multivariate approaches—e.g., a trade-off exists if trait loadings within a given principal component have opposite signs [42]. Unlike the one-dimensional leaf economics spectrum, the root economic space is hypothesized to be a bi-dimensional space, featuring orthogonal gradients for resource conservation and collaboration with symbionts [7,43]. Therefore, in addition to the analysis of PC loadings, examining the internal angles between loading vectors in an ordination plot provides valuable information related to trade-offs and orthogonality [8,30]. However, the expectations according to the RES framework were not completely met in our study system. Specifically, as expected, root economic traits associated with the collaboration (SRL and AD) and conservation (RTD and RN) gradients had opposite signs and most of them were aligned with different principal components (i.e., SRL and AD with PC1; RTD and RN with PC2) [30]. However, contrary to expectation, SRL and RTD, which are theoretically associated with different gradients [7], exhibited the largest angle (≈160°) and opposite signs along PC1, suggesting negative covariation. Conversely, RTD and AD, despite being associated with different gradients, displayed a sharp, acute internal angle (45°) far lower than the expected 90°, which suggests positive covariation. Therefore, we argue that collaborative and conservative gradients are not orthogonal at the intraspecific level in this species and that some of the expected trade-offs between root economic traits do not exist in chaya or occur in the opposite direction. This was also supported by univariate analyses. In these analyses, while RN and AD of domesticated plants were statistically different across soil origins, the opposite effect was not observed in the correlated traits according to the RES (RTD and SRL). This suggests that domesticated plants may possess the capacity to modulate individual root traits independently. This phenomenon may stem from a domestication-driven reduction in phenotypic integration documented in other crops [29], coupled with a relaxation of ecological trade-offs in resource-rich environments [12].

Another consequence of the observed deviation from the expected RES framework in gradient orthogonality as well as trait covariation is that, instead of a bi-dimensional economic space, the root economics in chaya may be better explained by a one-dimensional acquisition–conservation economic spectrum. While a recent literature review has confirmed the existence of an intraspecific bi-dimensional RES in several systems [30], there are noteworthy exceptions where no collaboration gradient exists [14]. Thus, root traits such as AD and SRL are sometimes interpreted as part of a single conservation-acquisition spectrum [44,45].

In this study, we assumed that AD is a reliable predictor of association with AMF, as this pattern has been reported in large multispecies studies [7,8]; however, this may not be the case for chaya. Therefore, future studies should assess whether AD serves as a reliable proxy for AMF association by directly quantifying root fungal colonization; otherwise, the existence of a collaboration gradient *sensu stricto* may be debatable in chaya. Another limitation of this study was that root economics could be influenced by the aerial portion of the plant—since photosynthates are translocated from leaves or shoots to roots [1,4]—yet above-ground traits related to the carbon economy were not measured here. While we minimized the influence of the light environment by using a common garden approach with uniform light exposure, we cannot rule out that other factors (e.g., differential resource allocation to leaves or shoots between wild and domesticated plants) may have influenced above-ground traits. Finally, although the common garden approach isolated the belowground environment as the main source of variation, field conditions in forest patches, home gardens, and orchards exhibit contrasting aboveground environments. Therefore, how above- and belowground environments interact to shape root economic traits remains an open question.

## 4. Materials and Methods

### 4.1. Study System

The study species was *Cnidoscolus aconitifolius* (Euphorbiaceae), a woody perennial shrub native to a region extending from southern Texas to Central America [46]. This species was domesticated by the Peninsular Maya, who named the crop “chaya” [47]. The target organ of growers are the leaves which are eaten after boiling [32,47]. While wild *C. aconitifolius* reproduces via seeds in natural habitats, domesticated plants are propagated clonally using stem cuttings [48,49]. Roots of 90-day-old, domesticated plants are heavier (1.28 ± 0.1 g), longer (30.82 ± 1.74 cm) and larger in volume (34.99 ± 2.7 cm^3^) than those of wild plants (0.70 ± 0.05 g, 23.81 ± 1.9 cm, 24.48 ± 2.7 cm^3^) (ANOVA, *p* < 0.01, *F*_1,124_ = 7.47–29.31; N = 138 plants; unpublished data). See Supplementary Materials for photographs of roots from wild and domesticated plants. Domesticated chaya has been cultivated in home gardens since its origins; however, it has recently been introduced into intensively managed orchards [33,49]. Soil management is predominantly organic in home gardens, whereas synthetic fertilizers are frequently used in intensive orchards [33]. Across home gardens, orchards, and forest patches, soil pH is consistently neutral (7.3 ± 0.1). However, organic carbon is significantly higher in forest patches (3.14 ± 0.32%) than in agroecosystems (home gardens: 2.48 ± 0.14; orchards: 2.35 ± 0.15%). Conversely, phosphorus content is higher in agroecosystems, particularly in home gardens (home gardens 3.04 ± 1.33 mg·kg^−1^; orchards 0.61 ± 0.25 mg·kg^−1^) than in forest patches (0.24 ± 0.15 mg·kg^−1^) [33]. Regarding the soil community of AMF or ectomycorrhizal fungi (ECM), we have identified 14 spore morphotypes; however, differences in community composition between soil origins were significant (PERMANOVA, F_2,42_ = 5.73, *p* < 0.01). Home gardens harbored the richest (q_0_ = 13.56 ± 0.09) and most diverse (q_1_ = 11.57 ± 0.15) community of AMF/ECM spores, followed by forest (q_0_ = 11.42 ± 0.19; q_1_ = 9.69 ± 0.17) and intensive orchards (q_0_ = 9.03 ± 0.17; q_1_ = 7.77 ± 0.15), while morphotype dominance and evenness were very similar among soil origins (see Supplementary Materials).

The study area was the Mexican portion of the Yucatan Peninsula. The climate is tropical sub-humid, with mean annual temperature ranging from 26 °C to 28 °C and an average annual rainfall of 1126 mm. The native forest vegetation consists of tropical dry forest dominated by *Lysiloma latisiliquum* (Fabaceae), *Piscidia piscipula* (Fabaceae), and *Bursera simaruba* (Burseraceae) [50]. Most species cultivated in the home gardens are native trees (e.g., *Brosimum alicastrum*, *Cordia dodecandra*, and *Talisia oliviformis*), whereas intensive orchards are dominated by non-native crop species (e.g., *Citrus × aurantium* and *Citrus × limon*) [51].

### 4.2. Soil and Plant Sampling

Topsoil samples of Leptosols were collected between March and December 2024 from three habitats with contrasting management types, forest patches, home gardens, and orchards (soil origins), in the municipality of José María Morelos, Quintana Roo (19°13′03″–20°11′24.36″ N, 88°23′29″–89°17′47″ W; 100–200 m a.s.l.). Bare soil samples (2 kg) were collected at a depth of 0–30 cm from 15 sites per soil origin (45 sampling sites in total). At each site, a five-point subsampling procedure was performed within a 300 m^2^ square plot. The five subsamples per plot were pooled into a single composite sample. A minimum distance of 2 km was maintained between sampling sites to ensure spatial independence. The composite soil samples were transported to the laboratory for use in the experiment described below (see the “Experimental design and data collection” subsection). Therefore, soil origin represents the cumulative effect of contrasting soil management.

Concurrently, stem cuttings were collected from two different but nearby localities with similar abiotic environments to avoid a home-field advantage effect. Domesticated plant cuttings were collected exclusively from the “mansa” cultivar, which is the most widely cultivated variety [32]. Stem cuttings of domesticated plants were collected from the locality of San Bernardo (20°38′09″ N, 89°57′10″ W) within traditional home gardens, while wild plant cuttings were collected from Maxcanú (20°16′01″ N, 89°47′13″ W) within disturbed tropical dry forest patches. The geographic distance between these two localities is approximately 5 km. Four stem cuttings (20–30 cm in length) were harvested from each of 30 wild and 30 domesticated adult plants (n = 240 cuttings in total). Plant selection was limited to individuals possessing at least three secondary branches. To prevent sampling closely related individuals, only one domesticated plant was sampled per home garden, and a minimum distance of ca. 6 m was maintained between sampled wild plants. All cuttings were immediately transported to a plant nursery located at our research facilities in Merida, Yucatan (21°01′18.3” N, 89°37′34.1” W). Because the study species is not under protection or listed as endangered, no permit was needed to collect the plant material.

### 4.3. Experimental Design and Data Collection

**Figure 4:**
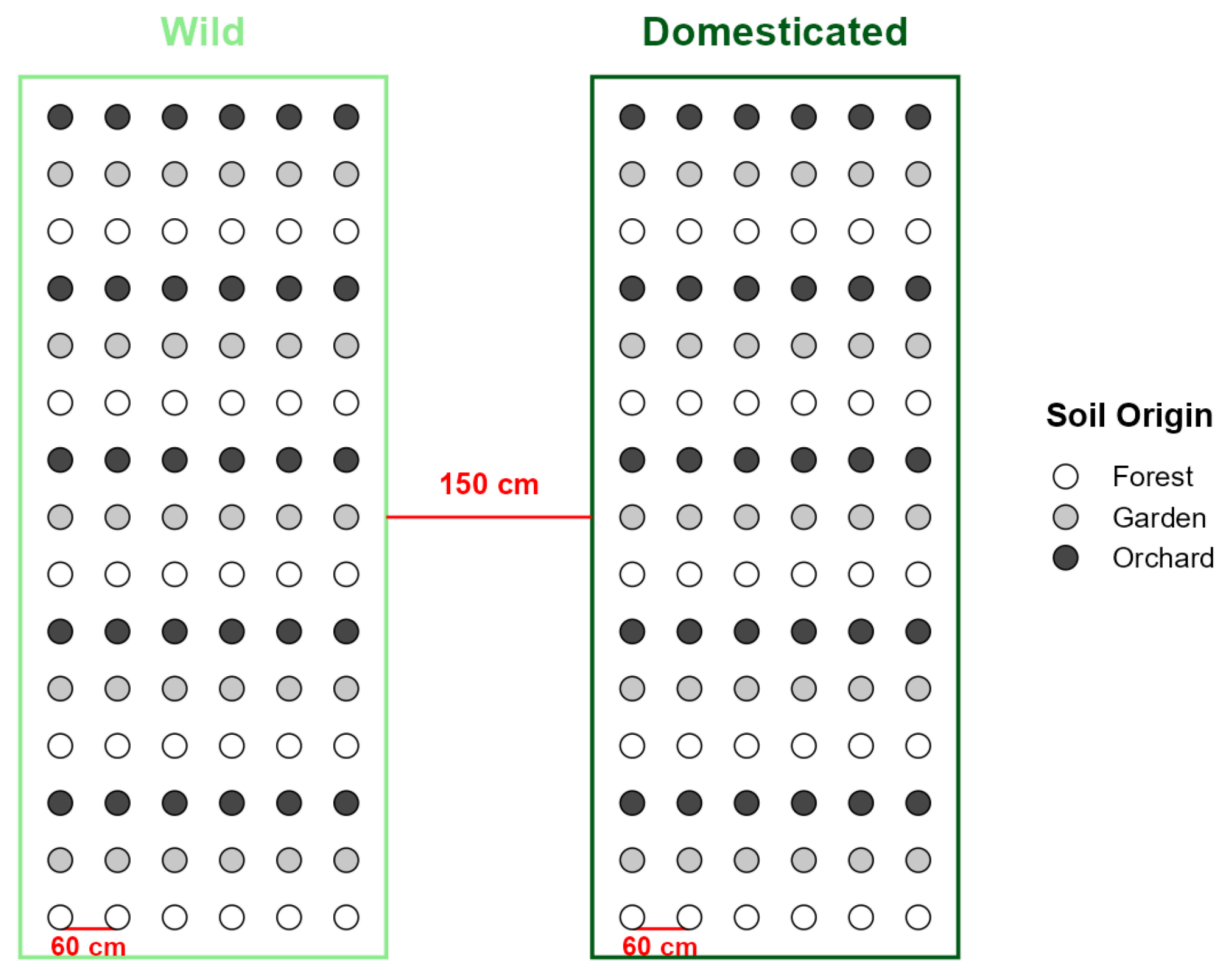
Diagrammatic scheme of experimental design. Circles represent root trainers, each containing a single cutting of either wild or domesticated chaya (*Cnidoscolus aconitifolius*) filled with soil from one of three origins: forest patches (Forest), home gardens (Garden), or intensive orchards (Orchard). The distance between plants was 60 cm. All plants were placed on bench tables in a nursery under full sunlight exposure. Wild and domesticated plants were assigned to separate tables spaced 150 cm apart. N = 180 root trainers and cuttings.

After an air-curing period of one week, three unrooted cuttings from each of the 60 mother plants sampled (30 wild and 30 domesticated) were planted individually in 180-L root trainers (10 × 7.5 × 29 cm). Cuttings were planted in a substrate consisting of ca. 2 kg of a 1:3 mix of soil and local limestone gravel per trainer. Gravel was added because the study species requires well-drained soils. Given the short time scale of the experiment, likely the main source of nutrients was the soil; any nutrient contribution from the gravel was negligible and uniform across treatments. The final sample size was 180 root trainers, each containing one cutting (30 cuttings × 2 plant varieties × 3 soil origins). The distance between experimental plants was ca. 60 cm within the nursery (common garden). All plants were watered uniformly twice a week throughout the three-month experimental period. No fertilization was applied, and plants were fully exposed to the sun. Plant survival was recorded weekly during the three months the experiment lasted. Dead plants were replaced with backup cuttings from the same mother plant only during the first 10 days of the experiment (Figure 4).

After three months, 138 surviving plants (21–24 plants per treatment combination) were harvested ensuring the recovery of the entire root system. Soil particles were carefully removed from the roots by washing them with tap water and the root system was separated from the stem using a scalpel. Intact roots were photographed using a professional digital camera alongside a graduated ruler as a size reference. Subsequently, the average root diameter (AD, mm), root volume (cm^3^), and root length (m) were measured using the SmartRoot plug-in of ImageJ 1.44 software [52]. Since all roots examined were young, first- to third-order roots with an AD less than 2 mm, they were all considered fine roots [53]. Photographed roots were oven-dried at 65 °C for 48 h, and their dry mass (g) was measured using an analytical balance (BOECO BAS 31 Plus, Hambug, Germany; accuracy: 0.01 mg). Using these data, root tissue density (RTD) and specific root length (SRL) were calculated. RTD (g·cm^−3^) was calculated as root dry mass divided by root volume, whereas SRL (m·g^−1^) was calculated as root length divided by root dry mass. Finally, a sample of approximately 0.2–0.5 g of dry root tissue was selected and finely ground to measure root N content (RN, %) using an elemental analyzer (FlashEA 1112, Thermo, Milan, Italy). For detailed procedures to measure and interpret the above-mentioned root traits, see Cornelissen et al. [54] and Freschet et al. [55].

### 4.4. Statistical Analyses

Root trait variation was first graphically assessed using boxplots, and the coefficient of variation (CV; defined as the ratio of the standard deviation to the mean in percentage) was calculated for each treatment combination. Linear mixed-effects models were used to assess the effect of domestication status (wild vs. domesticated), soil origin (forest, home garden, and orchard), and their interaction on the four root traits of the RES framework (AD, SRL, RN, and RTD). In all cases the mother plant (i.e., the genetic family) was included as a random factor. When soil origin was statistically significant, pairwise post hoc comparisons were conducted using estimated marginal means and *p*-values were adjusted using the Tukey–Kramer honest significant difference (HSD) test. When a significant interaction was detected, a simple effects analysis was conducted followed by a visual inspection of interaction plots to evaluate the specific effects of one factor at each level of the other. To assess the plastic response of wild and domesticated plants across soil origins, the plastic index (PI) outlined by Valladares et al. [56] was calculated as follows: PI = Maximum mean-minimum mean/maximum mean. The PI ranges from 0 to 1 and is widely used, thereby improving data comparability. Visual inspection and diagnostic tests of model residuals confirmed that the assumptions of normality and homoscedasticity were met in all cases.

Trait covariation was assessed using principal component analysis (PCA) to obtain the loadings of the first two principal components. To assess the RES, we followed the approach outlined in the most recent review on the topic [30] based on the relative orientation of loadings vectors in the PCA biplots. When SRL and AD loaded in opposite directions (internal angle of ca. 180°) on a principal component axis, it was considered support for a collaboration gradient. On the other hand, support for a conservation gradient was confirmed when RTD and RN showed opposite loadings (ca. 180°). Full support for the RES concept was granted only if both gradients were orthogonal to each other within the PCA space (internal angle of ca. 90°). To quantitatively address orthogonality, the internal angles of trait vectors (loadings) from the PCA biplot were calculated [8,30]. Internal angles for AD, SRL, RN, and RTD were computed using the arctangent function of their coordinate loadings. Since a relevant topic in this paper was gradient orthogonality, we used an unrotated PCA [8,30]. The matrix used in the PCA had some missing values for RN, given that in some samples the small amount of dry root tissue collected was below the detection limit of the elemental analyzer. Therefore, before PCA, missing values were filled using a soft imputation algorithm.

All statistical analyses were performed in R software version 4.5.1 [57]. Packages lme4, emmeans, factominer, and softImpute, as well as factoextra and ggplot2, were used. Raw data are available as Supplementary Materials.

## 5. Conclusions

This study demonstrates that the effect of domestication on some of the core traits of the RES is contingent upon soil origin. That is, rather than acting as a simple, isolated driver, domestication has interactively modulated root economics by significantly enhancing phenotypic plasticity in domesticated chaya. Under the nutrient-luxury conditions of agroecosystems, domesticated plants exhibited a pronounced capacity to decouple and modify individual root traits independently—such as increasing metabolic investment (high RN) or autonomous exploitation (low AD) depending on the specific soil context—whereas wild plants remained phenotypically canalized. Furthermore, the results also reveal that the expected trait covariation pattern and gradient orthogonality were only partially met in this study system. Overall, the findings highlight that incorporating phenotypic plasticity and trait-by-environment interactions is essential to fully understand how domestication reconfigures the belowground economics of domesticated plants.

## Figures and Tables

**Table 1:** Coefficients of variation (%) for four functional root traits associated with the root economics space in wild and domesticated chaya plants (*Cnidoscolus aconitifolius*) grown in soils from three different origins: forest patches (Forest), homegardens (Garden), and intensive orchards (Orchard). Overall coefficients of variation for each variety (across all soil origins) and each soil origin (across both varieties) are included as row and column totals, respectively (in bolds). N = 138 plants.

			Origin		
Root Trait	Variety	Forest	Garden	Orchard	Total
AD	Wild	51.14	44.09	14.12	**43.05**
	Domesticated	27.41	105.52	48.78	**74.17**
	**Total**	**40.03**	**90.38**	**41.98**	
SRL	Wild	171.72	245.93	38.03	**256.30**
	Domesticated	120.32	47.78	47.92	**124.21**
	**Total**	**178.33**	**273.52**	**84.43**	
RTD	Wild	109.17	182.84	24.40	**127.94**
	Domesticated	74.78	54.12	270.17	**387.41**
	**Total**	**90.89**	**216.32**	**265.28**	
RN	Wild	56.63	38.23	36.82	**44.47**
	Domesticated	89.01	21.36	21.83	**63.38**
	**Total**	**78.33**	**30.49**	**55.32**	

Abbreviations: AD = average root diameter; SRL = specific root length; RTD = root tissue density; RN = root nitrogen content.

**Table 2:** Results of linear mixed models conducted to assess the effects of domestication (wild vs. domesticated), soil origin (forest, home gardens and intensive orchards) and their interaction (Domestication × Origin) on four root traits associated with the root economics space of chaya (*Cnidoscolus aconitifolius*). Fixed effects were evaluated using a Wald’s Chi-square (χ^2^) tests, and their effect sizes are presented as percentage of the explained deviance. For the random factor (genetic family), the proportion of total variance explained by the random intercept is reported.

Root Trait	Source of Variation	D.F.	Statistics	Explained Deviance (%)
AD	Domestication	1	χ^2^ = 5.67 *	2.41
	Origin	2	χ^2^ = 12.88 **	17.31
	Domestication × Origin	2	χ^2^ = 59.64 **	80.14
	Random factor			0.01
SRL	Domestication	1	χ^2^ = 0.86	1.00
	Origin	2	χ^2^ = 7.16 *	1.00
	Domestication × Origin	2	χ^2^ = 1.97	1.00
	Random factor			15.14
RTD	Domestication	1	χ^2^ = 0.01	0.29
	Origin	2	χ^2^ = 0.86	2.4
	Domestication × Origin	2	χ^2^ = 2.23	0.66
	Random factor			2.11
RN	Domestication	1	χ^2^ = 0.01	1.78
	Origin	2	χ^2^ = 0.86	3.45
	Domestication × Origin	2	χ^2^ = 32.23 **	89.97
	Random factor			0.63

* *p* < 0.05, ** *p* < 0.01. Abbreviations: D.F. = Degrees of freedom; AD = Average root diameter; SRL = Specific root length; RTD = Root tissue density; RN = Root nitrogen content.

**Table 3:** Mean values (±1SE) for four root economics traits of two varieties (Wild vs. Domesticated) of chaya plants (*Cnidoscolus aconitifolius*) grown in soils of three different origins: forest patches (Forest), home gardens (Garden), and intensive orchards (Orchard). Means for all treatment combinations as well as overall totals are shown in bolds. A phenotypic plasticity index (PI) for wild and domesticated plants and its difference (ΔPI) across all soil origins are also included. Different lowercase letters indicate significant differences between varieties within each soil origin, whereas different capital letters indicate significant differences between overall totals (for varieties in columns and soil origins in rows, respectively; Tukey’s HSD, *p* < 0.05). N = 138 plants.

		Soil Origin					
Root Trait (Units)	Variety	Forest	Garden	Orchard	Totals	PI	ΔPI
AD (mm)	Wild	0.46 ± 0.05 ^a^	0.67 ± 0.06 ^a^	0.60 ± 0.02 ^a^	**0.56 ± 0.03 ^A^**	0.37	0.48
	Domesticated	0.52 ± 0.03 ^a^	0.11 ± 0.02 ^b^	0.71 ± 0.07 ^a^	**0.47 ± 0.04 ^B^**	0.85	
	**Totals**	**0.47 ± 0.03 ^A^**	**0.40 ± 0.06 ^A^**	**0.66 ± 0.04 ^B^**			
SRL (m·g^−1^)	Wild	3.74 ± 1.40 ^a^	5.74 ± 3.01 ^a^	1.01 ± 0.08 ^a^	**3.61 ± 1.17 ^A^**	0.82	0.05
	Domesticated	1.65 ± 0.44 ^a^	1.61 ± 0.17 ^a^	0.22 ± 0.02 ^a^	**1.09 ± 0.16 ^A^**	0.87	
	**Totals**	**2.72 ± 0.75 ^A^**	**3.77 ± 0.16 ^A^**	**0.56 ± 0.07 ^B^**			
RTD (g·cm^−3^)	Wild	1.01 ± 0.24 ^a^	2.90 ± 1.13 ^a^	4.54 ± 0.25 ^a^	**2.76 ± 0.45 ^A^**	0.78	0.15
	Domesticated	1.18 ± 0.19 ^a^	0.68 ± 0.80 ^a^	9.79 ± 5.29 ^a^	**4.33 ± 2.08 ^A^**	0.93	
	**Totals**	**1.09 ± 0.16 ^A^**	**1.84 ± 0.61 ^A^**	**7.53 ± 3.01 ^A^**			
RN (%)	Wild	1.69 ± 0.36 ^a^	1.64 ± 0.19 ^a^	1.29 ± 0.16 ^a^	**1.53 ± 0.13 ^A^**	0.23	0.41
	Domesticated	2.08 ± 0.62 ^a^	1.42 ± 0.08 ^a^	3.96 ± 0.26 ^b^	**2.39 ± 0.26 ^A^**	0.64	
	**Totals**	**1.81 ± 0.37 ^A^**	**1.52 ± 0.08 ^A^**	**2.76 ± 0.35 ^A^**			

Abbreviations: AD = Average diameter; SRL = Specific root density; RTD = Root tissue density; RN = Root nitrogen content.

## Data Availability

Raw data are available as online Supplementary Materials.

## Supplementary Materials

The following supporting information can be downloaded at https://www.mdpi.com/article/10.3390/plants15182850/s1: S1: Root photographs, S2: Analysis of AMF spores in different soil origins, S3: Raw data.

## Abbreviations

The following abbreviations are used in this manuscript:

RES	Root economics space
AD	Average root diameter
SRL	Specific root length
RN	Root nitrogen content
