# Supplementary material for: Domestication Enhances the Plastic Response of Root Economic Traits to Soil Variation in the Perennial Crop Cnidoscolus aconitifolius
Source: Plants (Basel). 2026 Sep 18;15(18):2850. doi: 10.3390/plants15182850 (PMC13611598; doi:10.3390/plants15182850)

Supplementary material: Analysis of the arbuscular mycorrhizal fungi (AMF) spore community in soils from three different origins: forest patches, homegardens, and intensive orchards in the Yucatan Peninsula.

Table S1. Diversity profiles (q_0, q_1, q_2) and evenness ($E_{1,0}$) of AMF spores. Data are mean values ± 1SE. Total sample size was n=45 (15 soil samples x 3 soil origins [forest patches, homegardens and intensive orchards]). With the exception of $E_{1,0}$ (calculated as the ratio of q_1 to q_0), the statistics represent the result of one-way ANOVAs. Superscript letters indicate statistically significant differences assessed using Tukey's HSD post-hoc tests.

Metric	Statistics	Forest	Garden	Orchard
q_0	$F_{2,42}$=329.19**	11.42 ± 0.19[a]	13.54 ± 0.09[b]	9.03 ± 0.17[c]
q_1	$F_{2,42}$=167.47**	9.69 ± 0.17[a]	11.57 ± 0.15[b]	7.77 ± 0.15[c]
q_2	$F_{2,42}$=112.08**	1.13 ± 0.03x10^{-1} [a]	1.11 ± 0.03x10^{-1} [b]	1.18 ± 0.07 x10^{-1}[c]
$E_{1,0}$		0.85	0.85	0.86

**$P < 0.01$

Figure S1. Rank-abundance curves for the arbuscular mycorrhizal fungi (AMF) spore community in soils from three different origins: forest patches (Forest), homegardens (Gardens) and intensive orchards (Orchard). m1-m14 indicates distinct spore morphotypes.

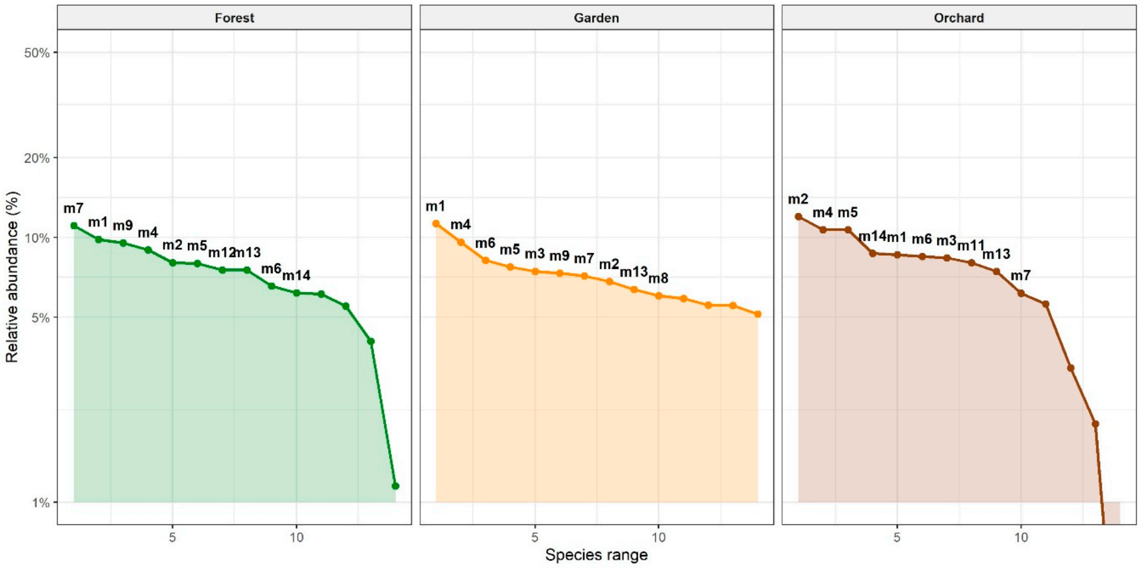

Supplement: Supplementary file 1 [file plants-15-02850-s001.zip › Supplementary_Material_S2.pdf]
